# Chemical Shrinkage of Low Water to Cement (w/c) Ratio CEM I and CEM III Cement Pastes Incorporating Silica Fume and Filler

**DOI:** 10.3390/ma14051164

**Published:** 2021-03-02

**Authors:** Judy Kheir, Benoît Hilloulin, Ahmed Loukili, Nele De Belie

**Affiliations:** 1 Magnel-Vandepitte Laboratory for Structural Engineering and Building Materials, Ghent University, Technologiepark Zwiijnaarde 60, B-9052 Ghent, Belgium; judy.kheir@ugent.be; 2 Institut de Recherche en Génie Civil et Mécanique (GeM), Ecole Centrale de Nantes, UMR-CNRS 6183, 1 Rue de la Noë, 44321 Nantes, France; benoit.hilloulin@ec-nantes.fr (B.H.); ahmed.loukili@ec-nantes.fr (A.L.)

**Keywords:** cement paste, chemical shrinkage, dilatometry method, ASTM C1608, silica fume, filler, paste thickness effect, w/c effect

## Abstract

Chemical shrinkage (CS) is the reason behind early age cracking, a common problem for concrete with low water to cement ratios (w/c < 0.35) known as Ultra-High- and High-Performance Concrete (U-HPC). However, to avoid the crack development initiated by autogenous shrinkage, a precise measurement of CS is required, as the values obtained can determine the correct amount of internal curing agent to be added in the mixture to avoid crack formation. ASTM C1608 is the standardized method for performing CS tests. In this study, recommendations are provided to improve the reliability of results obtained with this standard method, such as good compaction of samples and the use of superplasticizer (SP) for low w/c ratios (≤0.2). Cement pastes with CEM I and CEM III have been tested at different w/c ratios equal to 0.2, 0.3 and 0.4 with and without the addition of superplasticizer. CS results following ASTM-C1608 dilatometry showed that the presence of mineral additions such as silica fume and filler reduced the chemical shrinkage, while CS increased with increasing w/c. Low w/c ratio pastes of CEM III had slightly higher CS rates than CEM I, while the opposite was noticed at higher w/c. SEM images illustrated the importance of a careful compaction and SP use.

## 1. Introduction

Concrete is the most popular construction material, due to its easy transportation and low cost in addition to being a strong and resistant material. Its use dates back to 6500 B.C. in the Middle East, but concrete has evolved tremendously since then. While concrete as a building material has many advantages, there are also some challenges related to its use. One of these is the change in its volume, particularly during the hardening stage, which can lead to cracking, reduced durability, and aesthetics and functionality problems [1]. Early age cracking has been reported progressively over the past decade, especially in low water to cement ratio (w/c < 0.35) concrete i.e., HPC and UHPC and causes a decrease of durability in these types of concrete [2,3]. These low w/c concretes are being increasingly used in recent years for their remarkable and high mechanical strength properties. They contain supplementary cementitious materials (SCM) that dramatically affect the chemical shrinkage, autogenous shrinkage, internal humidity and stiffness of the matrix [4,5,6]. Shrinkage at early age has significant contribution to the overall shrinkage of cementitious materials, thus increasing cracking [7]. These early cracks are mainly linked with chemical (including autogenous) shrinkage of concrete [8]. Concrete is generally, at very early ages, well cured so the capillary actions related to drying shrinkage can be regarded as negligible [9]. 

Chemical shrinkage is defined as the absolute internal volume reduction of the hydration products of binders compared to the volume of initial components caused by the hydration reaction of cement. A desaturation of pores is then caused after the setting of the cementitious material [10]. It has an important contribution to the early concrete volume change [9]. Autogenous shrinkage is the bulk volume change of a sealed cementitious specimen under isothermal conditions (not subjected to external forces) [11]. If mortar and concrete are considered to consist of two phases (aggregate/sand particles and cement paste matrix), it is then important to study the shrinkage of cement pastes for a better understanding of that of concrete/mortar. 

Few studies can be found in the literature that deal simultaneously with chemical and autogenous shrinkage of the same cement pastes. Sant et al. [12] studied autogenous and chemical shrinkage measurements on the same cement paste with a w/c = 0.3. They showed that when the paste is still in the fluid phase, both AS and CS behaved similarly, since the fluidity of the paste does not allow the pores to be emptied. Nevertheless, these measurements diverge at a certain point around 7 h after water-cement contact. This point was defined as the setting point (where the paste develops its rigid skeleton, the start of the hardening phase) based on Vicat tests. Around that time, the shrinkage measurements diverge and AS is lower than the chemical shrinkage as the newly formed paste resists the volume change. Lu et al. [13] studied the effect of different SCMs such as fly ash, silica fume, and blast furnace slag (BFS) on the autogenous shrinkage of blended cement pastes with w/c = 0.3 and 0.4. The measurements of chemical shrinkage and autogenous shrinkage were performed in addition to internal relative humidity, compressive strength, and final setting time. CS of blast furnace slag cement developed the fastest at low w/c ratios as BFS cement pastes have finer pore structure than Portland cement (PC) pastes. Fly ash blended pastes had developed the lowest shrinkage curves. Fly ash has a lower reactivity than PC, which leads to a slower hydration and a slower decrease in the internal relative humidity thus smaller shrinkage [13,14].

The main goal of any chemical shrinkage test is to determine the change in volume that happens due to hydration reactions. It is generally measured by the amount of water that is being absorbed by saturated cement pastes [15]. CS tests allow water to enter the newly formed cementitious structure and measure the total volume change (including inner porosity) of the cement paste; the system can be described as an open system. On the contrary, AS tests do not allow water to re-enter the structure and refill the vapor-filled pores, because it is a closed and sealed system; these tests measure only the external volume change of the cementitious specimen. Hence, this causes the difference between the AS and CS results [12]. Over the last decade, chemical shrinkage tests were used to quantify different parameters in the cementitious materials. CS measurements were used by Powers [16] as indicator/index of the reaction progress; they were also used to be related to the compressive strength development as shown by Geiker and Knudsen [17,18] in addition to their use as indicators for the autogenous shrinkage extent that might occur in concrete [19]. More recently, CS measurements were used to identify the amount of internal curing materials needed in the mixture to avoid cracking [10]. Three methods have been reported in the literature to perform CS tests which are known as dilatometry, pycnometry and gravimetry. Dilatometry measures the volume change of the absorbed water while the other methods measure the change in buoyancy of the immersed cement paste sample. According to Geiker, dilatometry and gravimetry give comparable results [17], in this study only the dilatometry method was used. Several experimental difficulties can be found in these test methods that can result in false results: the thickness of the specimen and the chemistry of the water to be added on top, influence the results. For low water to cement (w/c) ratios below 0.4, the specimen will develop a lower pore connectivity, therefore, water can no longer travel to the vapor-filled pores created by CS [17,19].

While the standard procedure given by the American Society for Testing and Materials (ASTM) C1608 [20] seems satisfactory for measuring chemical shrinkage in cement pastes, difficulties to obtain a homogeneous and compacted paste in addition to spurious results, such as an overestimation of the CS, arise when dealing with low w/c mixtures. Therefore, in this study, chemical shrinkage tests were performed for different cement pastes according to the ASTM C1608 dilatometry method; pastes were prepared with CEM I and CEM III. These pastes were tested at different w/c ratios equal to 0.2, 0.3, and 0.4, with and without the addition of superplasticizer. The mineral additions used were silica fume and filler. Besides presenting novel insights regarding the CS testing procedure, the effects of mineral additions (slag, silica fume), filler and SP on CS of ternary and quaternary cement pastes are discussed.

## 2. Materials and Methods

### 2.1. Materials

**Figure 1:**
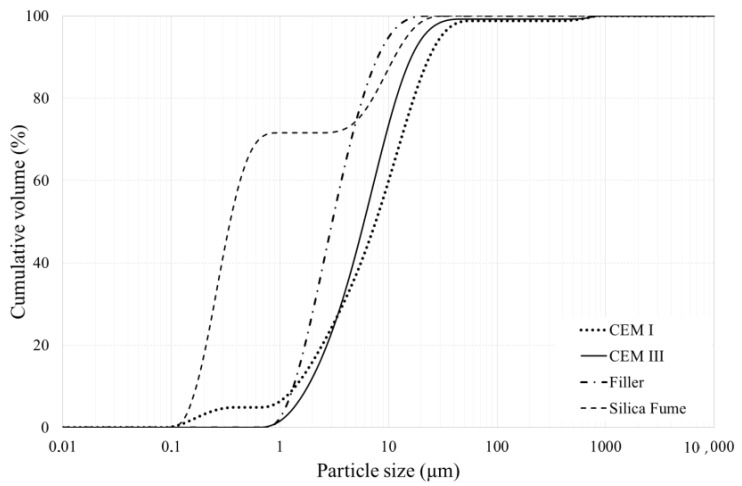
Particle size distribution of CEM I, CEM III, silica fume, and filler as determined by laser diffraction.

Two types of cement were used, a blast furnace slag cement CEM III/A 52.5 R Variodur 40 and a Portland cement CEM I 52.5 R, both produced by the company Dyckerhoff (Wiesbaden, Germany for the use in ultra-high- and high-performance concrete. Different cement paste combinations were tested with water to cement (w/c) ratios equal to 0.2, 0.3 and 0.4. The combinations contained filler, silica fume and superplasticizer; a lime filler called Betofill VK 50 with a density of 2700 kg/m^3^ along with a dry undensified silica fume powder (Microsilica 940U) with a density of 2200 kg/m^3^ were used in the mixture. Particle size distribution curves of the materials can be found in Figure 1, the measurements were performed according to the standardized Laser Diffraction method ISO 13320 [10] on a Mastersizer 2000E (Mastersizer, Malvern, UK) with a Hydro 2000SM wet unit (50–120 mL) (Malvern, UK).

The superplasticizer Sika ViscoCreteUHPC2 (Sika AG, Baar, Switzerland) with a density of 1.08 ± 0.02 kg/liter and 40% of active materials was used with a dosage of 1.1% by weight of cement. This value was chosen based on the amount added in the related HPC concrete composition under study [21] to aim at a slump flow of 700 mm (SF2). De-aerated water was used to fill the vials in the test. 

### 2.2. Cement Pastes Composition 

Different cement paste compositions were prepared as shown in Table 1. CEM III was combined with silica fume (SF), filler (F) and/or superplasticizer (SP). Some pastes with CEM I were also made for comparison. The pastes were prepared at w/c = 0.2, 0.3 and 0.4. The pastes with the mineral additions contained the representative amounts of binder used in the HPC, which served as a basis for the current study. The amount of silica fume used in the paste was equal to 19.76% by weight of cement and 23.79% for the filler according to the amounts used in the desired HPC concrete mixture. Furthermore, cement pastes were also tested with the incorporation of each mineral addition separately to see the effect of every mineral addition by itself on the shrinkage of the paste. In the following table the nomenclature of samples goes as follows: the first part belongs to the cement type (I for Portland cement CEM I 52.5 R or III for the CEM III/A 52.5 R) followed by SP for samples with superplasticizer, the second part is for the additives (SF or F for silica fume or filler and SF + F for the combination of both) and the last part is for the value of w/c (0.2, 0.3 or 0.4).

### 2.3. Mixing Protocol

The pastes were mixed as follows (following the same mixing protocol used for the HPC): first the dry compounds were mixed for 30 s in a Matest-E093 mortar mixer at low speed (140 rpm) and then water was added. SP was added at 60 s and then mixing at low speed was continued until the fifth minute. After these five minutes, one minute of a high-speed mixing (285 rpm) was used followed by a minute of scraping and resting. At the seventh minute, mixing at high speed was continued for 3 min. The time needed for the whole mixing cycle for each paste was equal to 10 min. 

### 2.4. Chemical Shrinkage 

**Figure 2:**
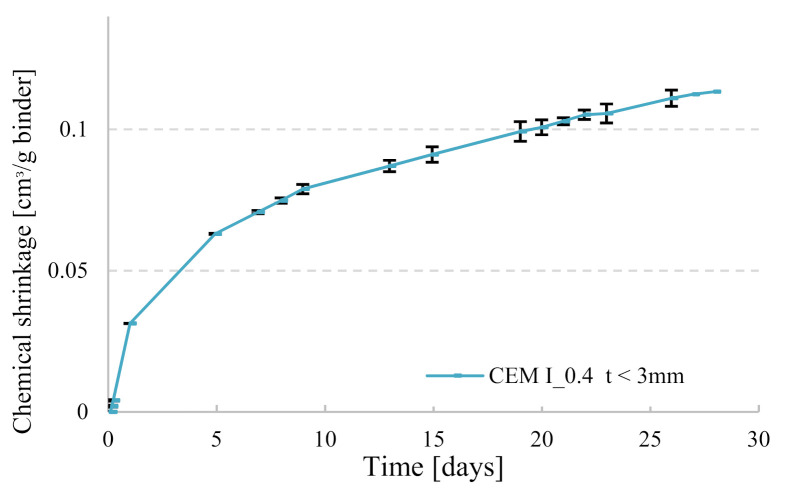
Representation of the repeatability of chemical shrinkage (CS) measurements (error bars represent the maximum and the minimum value taken from the sample replicas).

**Figure 3:**
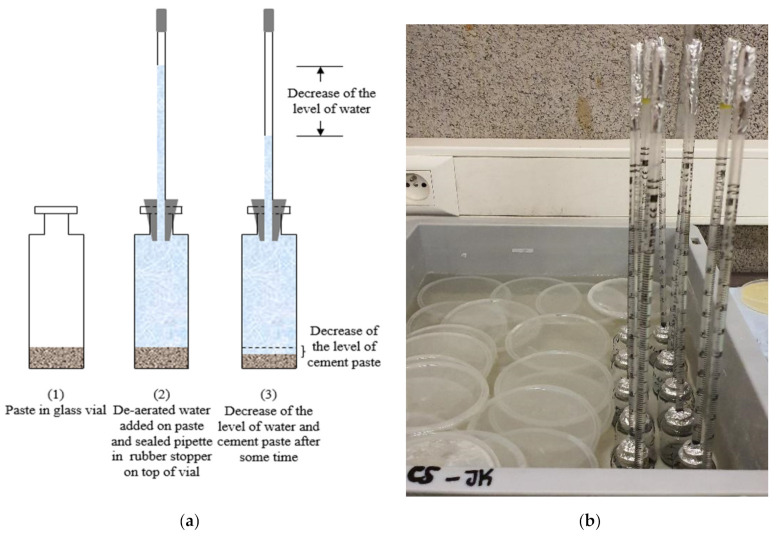
Test set up for chemical shrinkage: (**a**) schematic diagram, (**b**) samples in water bath at constant temperature (during the test the water bath is covered by lids to avoid evaporation).

Chemical shrinkage was measured according to ASTM C1608 [20]. Glass vials of 12 mL were filled with around 16 g to 18 g of paste (depending on the thickness desired as explained hereafter) and vibrated for at least 15 s. De-aerated water was slowly poured until the top of the vial. A pipette was introduced into a rubber stopper and placed on top of the glass vial; the top of the pipette and the top of the vial (pipette-rubber stopper-glass interface) were sealed with an aluminum tape to avoid water evaporation from the specimen. Two replicas were used for each paste composition, the repeatability between the replicas for one paste is represented in Figure 2. All other pastes had similar average difference between replicas, but error bars were not shown on curves in order to have clear graphs. All samples were put in a box of water filled until the neck of the glass vials and positioned in a climate-controlled room at 20 °C ± 2 °C and 60% relative humidity (Figure 3). To ensure that water evaporation did not take place, the mass of each sample (vial + paste, stopper and pipette) was recorded before introducing it into the bath of water, then after 24 h and a last measurement was taken at 28 days at the end of the experiment. If the difference between the initial and the final masses for a given sample was greater than 0.02 g, that specimen’s chemical shrinkage data were discarded. For the specimens with w/c = 0.2, the thickness of the paste in the tube was equal to 3 mm or below, a value recommended by ASTM for w/c < 0.4. In a thick paste layer, C-S-H gel formed can create a lot of inclusive pores where external water cannot penetrate. In order to ensure that these pores will always have access to water uptake from the surrounding, a small thickness less than 3 mm should be maintained. In this way, de-percolation is avoided, and chemical shrinkage behavior can be monitored by following the water decrease in the pipette. For specimens with w/c = 0.4, two groups with different thicknesses were investigated to quantify its effect on the performance of the chemical shrinkage test. The thickness of the first group was maintained between 5 mm and 10 mm (ASTM recommendation for w/c ≥ 0.4), whereas for the second group less than 3 mm was taken, in order to compare the results with low w/c pastes. 

After setting, chemical shrinkage can be measured while water is being sucked into the paste sample refilling the emptied pores, the measurements were followed up to 28 days. The zero point of chemical shrinkage was set to 90 min after the contact of water and cement (60 min after immersion in water), to allow for temperature equilibrium to be reached within the water bath while remaining ahead of the setting point of the cement paste.

The chemical shrinkage CS is calculated as the measured mL of absorbed water per gram of specimen binder. The mass of the powder in the tube is given by [1,2]:

(1) $$M_{\mathrm{binder}}=\frac{\left(M_{\mathrm{vial}+\mathrm{paste}}-M_{\mathrm{vialempty}}\right)}{\left(1+\frac{w}{b}\right)}$$

where: $M_{\mathrm{binder}}$ = mass of the binder paste in the vial (g), $M_{\mathrm{vial}+\mathrm{paste}}$ = mass of the glass vial plus the added paste (g), and $M_{\mathrm{vialempty}}$ = mass of the empty glass vial (g).

Thus,

(2) $$\mathrm{CS}\;\left(t\right)=\frac{\left[h\left(t\right)-h\left(90\;\min\right)\right]}{M_{\mathrm{binder}}}$$

where: CS (t) = chemical shrinkage per unit mass of binder paste at time t (t ≥ 90 min) (mL/g binder), h(t) = water level in pipette at time t (mL), h(90 min) = initial water level in pipette taken as zero point of chemical shrinkage (mL).

### 2.5. Scanning Electron Microscopy (SEM)

Scanning electron microscopy (SEM) characterization was used to assess the microstructure of cement pastes and contribute to identifying the homogeneity of the mixtures, pores, and the evolution of the matrix hydration reaction. Specimens taken from the vials were fragmented into small parts of only few millimeters at the ages of 14 and 28 days, they were introduced in a small circular plastic mold and then put in a vacuum chamber for 2 h to empty the pores. After two hours, epoxy was poured into the mold covering the samples. These specimens were then introduced in a vacuum chamber that was evacuated by a pump to remove entrapped air from the epoxy for about 1 h and to impregnate the specimens. Later, the mold containing the epoxy-coated specimens was put in an oven at 40 °C for at least 24 h. The impregnation is followed by careful polishing and grinding. Each sample was grinded on 500, 1200, and 2400 grit discs for 1.5 min on each disc, followed by final polishing performed using 3 µm, 1 µm and 0.5 µm diamond pastes with 4 min on each wheel (ethanol was used as lubricant). Finally, these samples were carbon coated to avoid charging during SEM imaging and were ready to be introduced in the microscope JEOL JEM 1400plus (JEOL, Tokyo, Japan) for microstructure analysis. A magnification of 1000 was used for all samples. 

## 3. Results and Discussion

### 3.1. Duration of Testing Period

**Figure 4:**
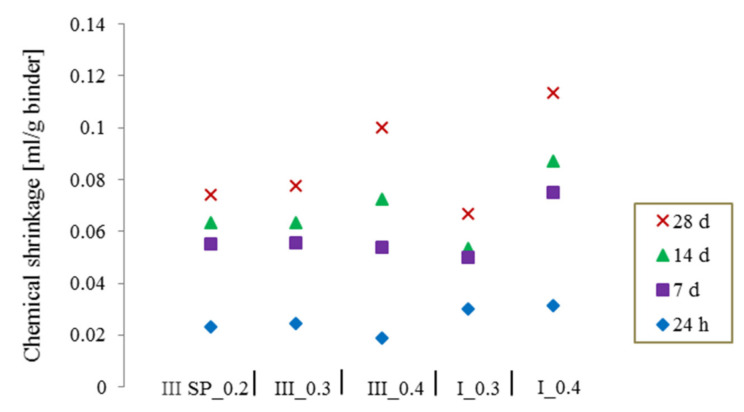
The increase of the chemical shrinkage over time for CEM I and CEM III.

The measurements were followed up to 28 days to make sure that most of the cement hydration process has occurred, the average value of CS was represented considering a 5 mm thickness for w/c = 0.4 and a thickness smaller than 3 mm for specimens with w/c = 0.2 and 0.3, according to the standard recommendations. In Figure 4, the value of the chemical shrinkage at different ages can be seen for the two different types of cement at different w/c ratios. Independently from the choice of w/c this value increased with increasing time. If the test is followed only until early ages, i.e., 24 h, 7 days, or even 14 days it may result in an underestimation of the shrinkage and therefore the amount of water needed for the internal curing of concrete. Therefore, it is highly important to allow a maximum hydration of the cement particles to obtain reliable values of total CS without falling into an underestimation of the amount of materials needed for internal curing.

### 3.2. Effect of Superplasticizer on Chemical Shrinkage of Cement Pastes

**Figure 5:**
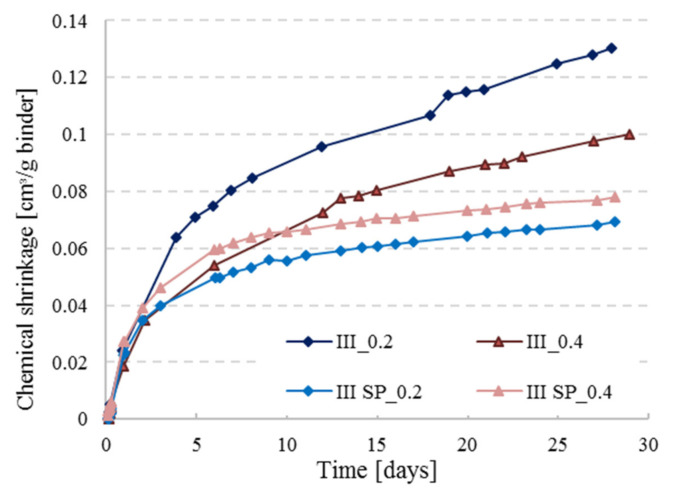
Chemical shrinkage behavior of CEM III pastes with and without superplasticizer at w/c = 0.2 and 0.4.

**Figure 6:**
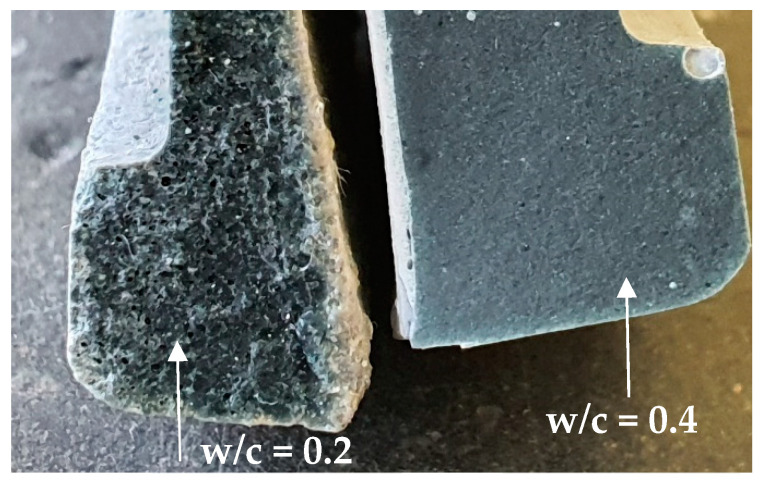
Cross-section of a cement paste with w/c = 0.2 (**left**) and a cement paste with w/c = 0.4 (**right**) at 28 days.

In Figure 5, the CS measurements for CEM III are presented for the reference pastes with and without the addition of superplasticizer (SP) at w/c = 0.2 and 0.4 (pastes inside the vials were kept at a thickness less than 3 mm). The represented curves are the average of two replicates for each paste. A clear difference can be observed between the two sets of curves, CS values are smaller with the use of SP for both w/c ratios. The difference is more pronounced with w/c ratio of 0.2. Superplasticizers are known for enhancing the workability of concrete and are often used in high performance concrete with low w/c ratio. Zhang et al. [22] stated that low w/c pastes (0.2 and 0.3) will lead to complications in preparing a homogeneous and fully compacted paste. Even though samples of that study [22] were placed twice under vacuum, there were still ambiguous results resulting from the presence of entrapped air bubbles. This could also explain the lower chemical shrinkage curves for the SP_0.2 set in Figure 5, because the paste without SP was not well compacted and clearly has more pores where water goes in slowly through time (see Figure 6). Thus, the water level in the pipette is decreasing due to this behavior but is interpreted as being chemical shrinkage. The paste layer thickness is reduced in order to avoid bad compaction, but nevertheless an erratic pore connectivity cannot be completely avoided. With the addition of SP, the mixture can be well compacted, so this problem does not occur. In the low w/c scenario, the gap between the curves is way more pronounced than for the higher w/c ratio because of the plasticizing effect discussed above.

**Figure 7:**
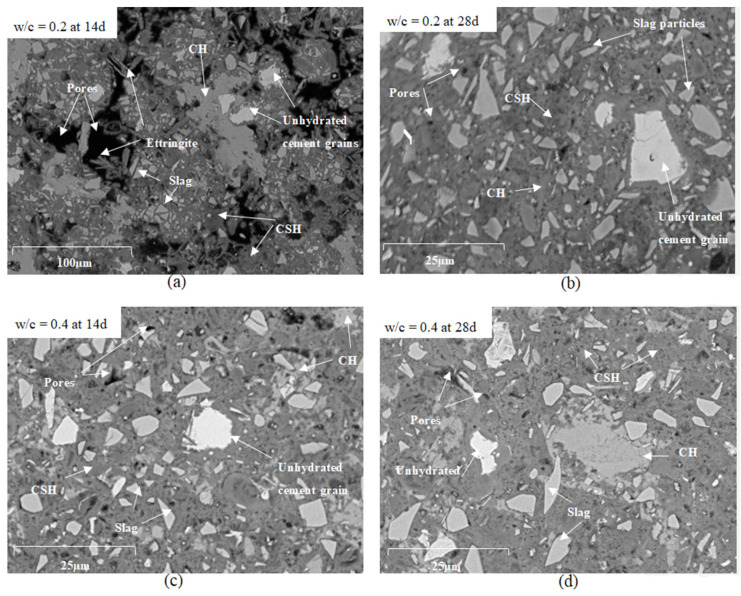
SEM images for cement pastes (**a**) with w/c = 0.2 (CEM III_0.2) at 14 days, (**b**) w/c = 0.2 (CEM III_0.2) at 28 days, (**c**) w/c = 0.4 (CEM III_0.4) at 14 days, and (**d**) w/c = 0.4 (CEM III_0.4) at 28 days.

The addition of superplasticizer is primordial to low w/c cement pastes for this test, which can be very well illustrated under microscopy, see Figure 7.

The microstructures of reference cement pastes with w/c = 0.2 and 0.4 (CEM III_0.2 and CEM III_0.4) were investigated at the ages of 14 and 28 days by scanning electron microscopy (SEM). As illustrated in Figure 7 fewer pores are present in pastes with w/c = 0.4 that are presented as black spots on the images, and less unhydrated cement grains, more calcium-silicate-hydrate C-S-H and calcium-hydroxide C-H as compared to pastes with w/c = 0.2. A large amount of ettringite and CH were observed in the microstructure of the reference paste prepared using w/c = 0.2 at 14 days (Figure 7a) due to the increased porosity in the paste that provided the space for the development of CH and ettringite crystals owing to their expansive nature [23]. Large pore connectivity resulting from poor compaction can be observed at 14 and 28 days of age. If pastes with w/c = 0.2 without SP were to be considered, these large pores will absorb higher amounts of water placed above the sample. In that way, the reading of the water level on the pipette will mistakenly lead to an overestimation of the actual chemical shrinkage. If chemical shrinkage tests are being used for providing the right amount of internal curing agents, the experiments should be carried out and interpreted with caution.

### 3.3. Effect of Paste Thickness on Chemical Shrinkage of Cement Pastes

**Figure 8:**
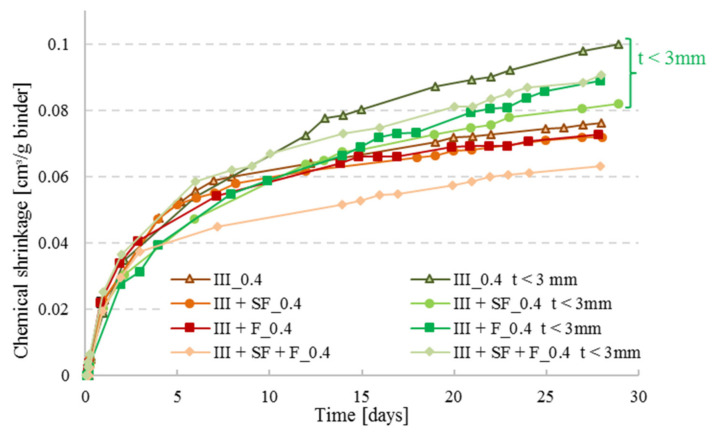
CS measurements of pastes with CEM III combined with silica fume (SF) and/or filler (F) at w/c = 0.4. Two sets were tested: one set at a thickness required by ASTM C1608 (t = 5 mm) and one set with thickness t < 3 mm.

As recommended by ASTMC1608, paste thickness for w/c ≥ 0.4 should be between 5 and 10 mm, whereas for lower w/c it should be maintained at < 3 mm. Boivin et al. [19] studied the effect of paste thickness on the CS of low w/c pastes, and found that it is more related to the water travelling inside the material rather than the chemical shrinkage itself; this was observed in this study as well, see Figure 8. At higher paste thickness, the kinetics of water penetration becomes slower than the kinetics of self-desiccation, therefore, the development of empty pores will not be completely compensated by the external water supply, and the measured shrinkage will be less than the real one [19]. For that reason and for the sake of comparison, it was chosen in this study to perform the series with w/c = 0.4 with a paste thickness of 3 mm.

### 3.4. Effect of Water to Cement (W/C) Ratio and Cement Type on Chemical Shrinkage

**Figure 9:**
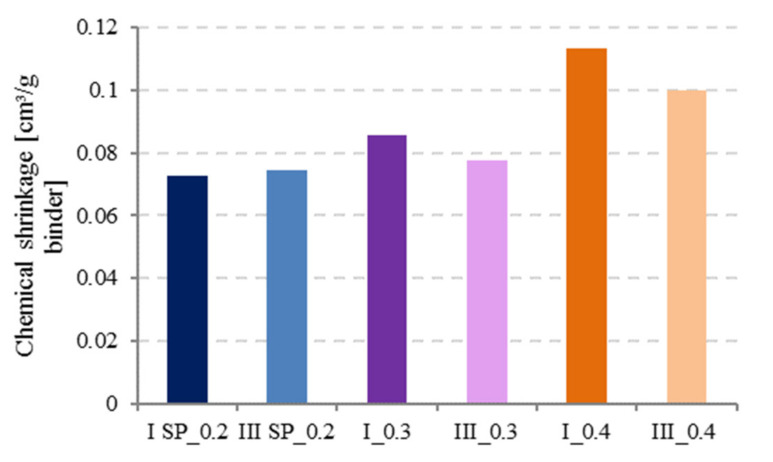
Effect of water to cement (w/c) ratio and cement type on chemical shrinkage at 28 d.

As illustrated by Figure 9, chemical shrinkage increased with increasing w/c ratio for constant paste thickness (t < 3 mm) and cement type as reported in literature, for all w/c ratios [2,24].

At low w/c ratios (0.2 and 0.3), it was observed that CEM III has slightly higher CS values than CEM I. Tianshi et al. [13] studied the CS of Blast Furnace Slag (BFS) and Portland Cement (PC) pastes with w/c = 0.3 and observed that BFS cement (CEM III) develops slightly higher chemical shrinkage than PC. This could be because at low w/c, hydration degree is lower, and since BFS cement has a finer pore structure compared to PC, hydrates supersaturation levels are reached more quickly in systems containing smaller pores and less water, leading to faster precipitation [19].

At w/c = 0.4, hydration proceeds faster in CEM I than in CEM III, the opposite phenomenon will occur and BFS is known to react later in time so higher CS values can be measured for CEM I. Nevertheless, the effect of the cement type cannot be considered to have a major influence on the CS values.

### 3.5. Effect of Mineral Additions on Chemical Shrinkage of Cement Pastes

**Figure 10:**
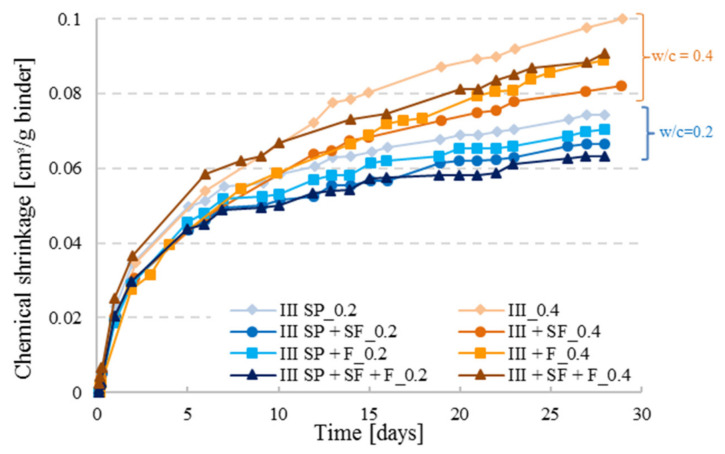
Chemical shrinkage results for CEM III with different paste combinations including silica fume (SF) and filler (F) for w/c = 0.2 with superplasticizer (SP) addition and 0.4 (paste thickness t < 3 mm).

As illustrated in Figure 10, the reference curves with the highest amount of CEM III cement lead to higher CS values as opposed to the ternary and quaternary cement pastes. The CS of CEM III cement pastes incorporating filler was slightly higher than for the one with CEM III and SF for both w/c ratios although the filler content was higher than the SF content, which implies that a lower amount of cement was present in the specimens containing filler. However, filler is not a reactive product in the cement hydration process, it is a material that decreases the porosity by filling the small voids produced by the hydration of cement in high performance concrete (due to its fine grains compared to cement) to have a well-organized micro-structure for a better durability of concrete. Whereas the silica fume is a product that reacts in the hydration process; SF consumes part of the portlandite formed by the hydration of cement for further pozzolanic reaction. Hence, bonding between the particles is enhanced in the cement matrix and fewer voids will be present in the skeleton which results in a decrease of the chemical shrinkage [25]. As it was noticed, the addition of mineral additions such as filler or SF reduces the chemical shrinkage which was also found in literature [26]; a combination of both further reduced the CS as illustrated by CEM III SP+SF+F series with w/c ratio of 0.2. For the cement pastes with w/c = 0.4 a particular behavior was seen for the combination (CEM III + SF + F_0.4), where a fast hydration took place in the first 10 days. As discussed before, at higher w/c ratio, the degree of hydration is higher as well, and when reactive agents are added to the mixture, the case of SF, hydration might be faster which can result in a higher CS curve. With time, hydration will be limited for the combination pastes having lower cement quantities.

## 4. Conclusions

Chemical shrinkage (CS) is the reduction of internal volume of concrete mixtures due to cement hydration reaction. In this study, two types of cement (CEM I and CEM III) were tested according to ASTM C 1608 at different water to cement ratios (0.2, 0.3 and 0.4) with and without the addition of superplasticizer (SP), using silica fume and filler additions while varying pastes thickness. The following conclusions can be drawn:

- Adding SP to low w/c cement pastes (≤0.2) and a careful compaction of paste samples by proper vibration must be provided when using the ASTM C1608 procedure. SEM images showed that bad compaction and absence of SP result in large pore connectivity that highly affects the shrinkage results.
- When comparing the method for different w/c ratios, it is important to choose the same paste thickness for all samples. The effect of paste thickness does not have a direct influence on the CS but rather on the water transport inside the material.
- Increase of w/c ratio results in higher CS values because higher w/c ratio pastes have higher degree of hydration, and more water is present in the mixture for hydrating cement grains.
- Cement type does not play a major role in the behavior of CS, but BFS cements (CEM III) tend to have slightly higher CS values than Portland cements at low w/c ratios, while the opposite seems to be true at higher w/c ratios.
- Addition of mineral components reduces the CS of CEM III pastes, as it is the case for silica fume and filler, and a combination of mineral additions (silica fume + filler) sometimes reduces CS further.

## Figures and Tables

**Table 1:** Cement pastes compositions.

Samples	CEM I 52.5 R (g)	CEM III/A 52.5 R (g)	Water (g)	Silica Fume (SF) (g)	Filler (F) (g)	Superplasticizer (SP) (g)
III_0.2	-	900	180	-	-	-
III + SF_0.2	-	751.8	180	148.12	-	-
III + F_0.2	-	727	180	-	173	-
III + SF + F_0.2	-	627.05	180	123.52	149.23	-
III SP_0.2	-	900	174.06 *	-	-	9.9
III SP + SF_0.2	-	751.8	175.04 *	148.12	-	8.27
III SP + F_0.2	-	727	175.20 *	-	173	7.99
III SP + SF + F_0.2	-	627.05	175.86 *	123.52	149.23	6.89
III_0.4	-	450	180	-	-	-
III + SF_0.4	-	375.94	180	74.29	-	-
III + F_0.4	-	363.49	180	-	86.62	-
III + SF + F_0.4	-	313.4	180	61.93	74.68	-
I SP_0.2	900	-	174.06 *	-	-	9.9
I_0.4	450	-	180	-	-	-
I_0.3	600	-	180	-	-	-
III_0.3	-	600	180	-	-	-

* Water provided from SP is accounted for: mass of water changes according to the mass of SP used because SP contains 60% of water.

## Data Availability

The data presented in this study are available on request from the corresponding author.
